# Alpha and theta band activity share information relevant to proactive and reactive control during conflict‐modulated response inhibition

**DOI:** 10.1002/hbm.26486

**Published:** 2023-09-20

**Authors:** Charlotte Pscherer, Paul Wendiggensen, Moritz Mückschel, Annet Bluschke, Christian Beste

**Affiliations:** ^1^ Cognitive Neurophysiology, Department of Child and Adolescent Psychiatry Faculty of Medicine of the TU Dresden Dresden Germany; ^2^ University Neuropsychology Center Faculty of Medicine, TU Dresden Dresden Germany

**Keywords:** alpha power, EEG, LCMV beamforming, proactive control, response inhibition, theta power, time‐frequency analysis

## Abstract

Response inhibition is an important instance of cognitive control and can be complicated by perceptual conflict. The neurophysiological mechanisms underlying these processes are still not understood. Especially the relationship between neural processes directly preceding cognitive control (proactive control) and processes underlying cognitive control (reactive control) has not been examined although there should be close links. In the current study, we investigate these aspects in a sample of *N* = 50 healthy adults. Time‐frequency and beamforming approaches were applied to analyze the interrelation of brain states before (pre‐trial) and during (within‐trial) cognitive control. The behavioral data replicate a perceptual conflict‐dependent modulation of response inhibition. During the pre‐trial period, insular, inferior frontal, superior temporal, and precentral alpha activity was positively correlated with theta activity in the same regions and the superior frontal gyrus. Additionally, participants with a stronger pre‐trial alpha activity in the primary motor cortex showed a stronger (within‐trial) conflict effect in the theta band in the primary motor cortex. This theta conflict effect was further related to a stronger theta conflict effect in the midcingulate cortex until the end of the trial. The temporal cascade of these processes suggests that successful proactive preparation (anticipatory information gating) entails a stronger reactive processing of the conflicting stimulus information likely resulting in a realization of the need to adapt the current action plan. The results indicate that theta and alpha band activity share and transfer aspects of information when it comes to the interrelationship between proactive and reactive control during conflict‐modulated motor inhibition.

## Graphical abstract

Response inhibition is an important instance of cognitive control that can be complicated by conflicting perceptual information. We provide a detailed analysis of theta and alpha band activity in relation to functional anatomical structures that informs about the relevance of proactive and reactive control during conflict‐modulated motor inhibition.

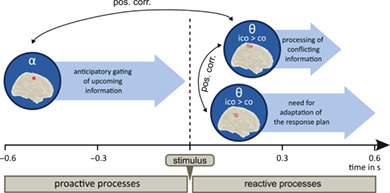

## 1 INTRODUCTION

Response inhibition is broadly investigated in cognitive neuroscience research and is of significant relevance to our daily lives (Diamond, 2013). Classic paradigms such as Go/Nogo tasks and Stop Signal tasks are used to investigate response inhibition (Huster et al., 2013), analyzing simple stimulus–response effects. However, the challenges we face in our daily lives are often more complex than withholding a response when one specific stimulus occurs. The trigger that elicits our responses is often composed of multiple perceptional stimuli, which can either imply the same or different reactions. Just imagine a traffic scenario: maybe a green light invites a pedestrian to cross the road, but simultaneously, the sound of a fast‐approaching car urges them not to do so. Thus, the pedestrian obtains interfering perceptional stimuli and has to inhibit the impulse of crossing the road, although the traffic light tempts him or her to do so. A prominent theory of perception‐action integration, the theory of event coding, suggests that perception and action—in our case, motor inhibition—are not isolated entities but are commonly represented (Hommel et al., 2001). Consequently, a variation on the perceptional (i.e., sensory) level, for example, in terms of a perceptional conflict, should directly affect the action level, that is, motor inhibition. Indeed, research has shown that multisensory interference complicates response inhibition and leads to increased false alarm rates (Chmielewski et al., 2016; Pscherer et al., 2019). There are two mechanisms to deal with interfering perceptional information: proactive control—referring to a sustained and anticipatory goal‐driven state, and reactive control—referring to a transient goal reactivation after an interfering event (Braver, 2012). The state of research on neurophysiological mechanisms underlying both types of cognitive control, as well as the derived hypotheses for the current study will be presented in the following.

### 1.1 Reactive control processes

At present, most of the research focuses on reactive control, i.e., brain activity between stimulus presentation and (withholding of a) response (Aron, 2011; Bari & Robbins, 2013; Raud et al., 2020). Nevertheless, even with reactive cognitive control, the underlying neurophysiological dynamics are far from being completely understood. This is particularly the case for oscillatory brain activity, and thus, a significant property of neural information processing (Buzsáki, 2006; Herrmann et al., 2016; Siegel et al., 2012; Ward, 2003). So far, increased theta activity accompanying successful response inhibition has been shown frequently (Chmielewski et al., 2016; Dippel et al., 2016, 2017; Huster et al., 2013; Liu et al., 2014; Müller et al., 2017; Pscherer et al., 2019, 2021, 2022; Vahid et al., 2018) and has been related to activation differences in the superior and medial frontal gyrus, the supplemental motor area (Dippel et al., 2017; Pscherer et al., 2019, 2021), as well as the temporo‐parietal junction (Dippel et al., 2016). Furthermore, when information is conflicting and increases the need for cognitive (inhibitory) control, medial frontal theta activity is elevated (Cavanagh & Frank, 2014; Cohen, 2014). Therefore, theta band activity should be increased during perceptual‐conflict modulations of response inhibition processes. However, alpha‐band activity is also likely to be modulated. This frequency band has been suggested to mediate processes related to the suppression of information in task‐irrelevant networks and thereby controlling the access to information relevant to behavior (Klimesch, 2011, 2012). In line with that, some studies reported that alpha activity in the lateral occipital (Wiesman & Wilson, 2020) and posterior parietal regions (Jiang et al., 2018) might reflect the detection and resolution of interference conflicts. Thus, alpha band activity should be higher when perceptual conflicts occur during response inhibition. Crucially, however, since both, theta and alpha band activity are thus likely to show functionally interrelated processes relevant to response inhibition modulated by a perceptual conflict, there should be a correlated pattern of theta band and alpha band activity during such perceptual conflict‐modulated response inhibition. However, it cannot be ruled out that alpha and theta activities might have different functionalities when it comes to response inhibition, which could affect the correlations in a hardly predictable way.

The first goal of the current study is to examine the interrelation of theta and alpha band activity related to reactive control. We define reactive control processes as within‐trial activity, i.e., activity occurring between stimulus presentation and (withholding of a) motor response. We analyze the pattern of possibly correlated theta and alpha band activity at source level in a data‐driven manner using a modified Go/Nogo task with interfering perceptual stimuli. For this purpose, we combine two different beamforming approaches (see Section 2 for details). The reason for analyzing and intercorrelating the source‐level theta and alpha band activity is that residual variance is decreased by the applied beamforming methods (Dippel et al., 2017; Van Veen et al., 1997), which increases the reliability of the correlational analyses. In addition, due to the spatial filtering properties of beamforming (Gross et al., 2001; Handy, 2009), the issue of spurious volume conduction effects affecting pre‐trial/within‐trial correlations of neural activity can be avoided.

### 1.2 Proactive control processes

Not only the reactive control processes will be examined, but also proactive neurophysiological processes. Previous studies have argued that pre‐trial activity, that is, processes that happen previous to stimulus onset may reflect proactive control (Adelhöfer & Beste, 2020; Adelhöfer et al., 2021). These recent studies have suggested that the neural activity evident in pre‐trial intervals, may be relevant to reactive inhibitory control taking place after stimulus presentation (Adelhöfer & Beste, 2020; Adelhöfer et al., 2021). Thus, it is essential to perform a more comprehensive analysis of neural processes involved in perceptual conflict‐modulated response inhibition, focusing on the pre‐trial period.

Especially regarding theta frequency band activity, pre‐trial activity has previously been associated with proactive control (Adelhöfer & Beste, 2020; Chang et al., 2017;Cooper et al., 2015; van Driel et al., 2015). In these studies, proactive control‐related activity was mostly measured after a cue predicting the upcoming stimulus (Cooper et al., 2015; van Driel et al., 2015). In the current study, we used a modified Go/Nogo task with interfering perceptual stimuli, which did not provide any cues. Yet, with an unequal trial ratio of 70% Go trials and 30% Nogo trials, a certain expectation of the participants toward the upcoming stimulus is built (Dippel et al., 2016). This may evoke attentional and working memory processes in preparation for the upcoming trial, which has also been associated with theta activity (Cavanagh & Frank, 2014; Hsieh & Ranganath, 2014; Hsieh et al., 2011; Jensen, 2006). Furthermore, the end of a trial (marked by the participant's response) can be seen as an implicit cue for the upcoming trial. Thus, in this study, we do not refer to an explicit cue‐based proactive control, but to a time interval in which participants prepare for the upcoming trial in a proactive manner. Previous studies used a similar approach (Adelhöfer & Beste, 2020; Adelhöfer et al., 2021). The brain state occurring before a stimulus might be influenced by attentional, motivational, and working memory processes. In the very few recent findings, proactive control‐related theta activity has been found in the ventromedial prefrontal cortex (Adelhöfer & Beste, 2020), in the pre‐supplementary motor area (pre‐SMA), and the supramarginal gyrus (Chang et al., 2017). Since the evidence is still sparse, we cannot predict the specific neuroanatomical regions associated with proactive control‐related theta activity in our study, but frontal and central regions are likely to be activated.

Given that the focus of the current study lies on perceptual conflict‐modulated response inhibition, and taking into account the dynamics during response inhibition processes (see above), also alpha activity in the pre‐trial interval is critical to consider for several reasons: In interference tasks, proactive (pre‐stimulus) alpha activity related to activity in the superior frontal cortex has been found and was suggested to inhibit the processing of irrelevant information (Suzuki et al., 2018). This finding is in line with the theoretical approach of Klimesch (2012), suggesting that alpha activity functions as an anticipatory inhibitory filter or attentional gating (Foxe & Snyder, 2011; Freunberger et al., 2008; Klimesch, 2012) that maintains relevant information activated and inhibits irrelevant processes. Alpha activity, therefore, is considered to play a significant role in information gating (Jensen & Mazaheri, 2010) and thereby also in theta‐frequency associated cognitive control processes (Beste et al., 2023). In a multisensory task (Misselhorn et al., 2019), frontal and parietal alpha activity has further been related to inter‐sensory orienting and attentional processes, which might also play a role in our task, which combines two senses (visual and auditory stimuli). Thus, we expect to find alpha activity in the pre‐trial time window in frontal and/or parietal regions.

### 1.3 Possible interrelation between proactive and reactive control processes

The impact of proactive control‐related theta activity on reactive inhibitory control has only recently become a topic of interest. Adelhöfer and Beste (2020) found that proactive control‐related theta band activity in the ventromedial prefrontal cortex is correlated with reactive control‐related theta band activity in the right inferior frontal cortex. In a study with a clinical sample, pre‐trial theta activity in right middle frontal regions was related to inhibitory control‐related theta activity in the cingulum (Adelhöfer et al., 2021). It seems crucial for the understanding of cognitive control to investigate the possible interrelations between proactive and reactive control processes in more detail because (a) theoretical considerations suggest that alpha and theta band activity might indeed be related to proactive control processes and (b) individual findings already suggest that an interrelationship between proactive and reactive control processes might exist. Recently, a close interaction between theta band and alpha band activity has been suggested, in that alpha band activity may act as a bottom‐up and top‐down attentional control for theta band activity (Beste et al., 2023). Thus, we are particularly interested in correlations between the two frequency bands.

Based on the presented background, our second hypothesis is that proactive control‐related theta and alpha activity correlates with reactive control‐related theta and alpha activity (associated with the perceptual conflict during inhibition in the modified Go/Nogo task). Therefore, we correlate pre‐trial activity with the activation differences between Nogo trials with versus without a perceptual conflict. Again, this is done in a data‐driven manner as described for the within‐trial dynamics. Specifically, within‐trial theta activation differences during Nogo trials will be correlated with within‐trial alpha activation differences during Nogo trials. Furthermore, pre‐trial theta activity will be correlated with pre‐trial alpha activity. Finally, pre‐trial alpha and theta activity will each be correlated with within‐trial theta and alpha activation differences during Nogo trials. By this means, correlations between two different frequency bands and two different time intervals (reflecting proactive and reactive control processes, respectively) can be investigated. A likely result could be that successful information gating (pre‐trial alpha activity) affects the processing of the following interference and inhibition effect (within‐trial alpha and theta activity).

The entire set of analyses in the pre‐trial and within‐trial periods and analyses on the interrelation between these periods in two different frequency bands will thus provide more comprehensive insights into the mechanisms underlying the modulation of inhibitory control processes by perceptual conflict. The study will provide insights into whether alpha and theta band activity share common aspects of information or code distinct aspects of information relevant to the proactive and reactive control processes during inhibitory control modulated by perceptual conflict.

## 2 METHODS AND MATERIALS

### 2.1 Participants

The sample consisted of *N* = 50 healthy, right‐handed participants (23 male, 27 female) randomly selected from a larger sample. We consider the sample size well‐powered since previous work from our group showed reliable effects using the same approach with a sample of *N* = 31 subjects (Adelhöfer & Beste, 2020). Our participants' age ranged from 18 to 40 years, with a mean age of 25.88 ± 5.63 years. IQ was estimated using a German multiple‐choice vocabulary intelligence test (MWT‐B, Lehrl, 2005) revealing an average IQ of 111.32 ± 12.15. The participants did not report any neurological or psychological disorders, which was corroborated by the ASR/18‐59 (Adult Self‐Report; Achenbach & Rescorla, 2003). Every participant provided written informed consent before data assessment and received financial compensation after completing the study. The local ethics committee of the TU Dresden approved the study procedures, which were conducted in accordance with the Declaration of Helsinki.

### 2.2 Task

We used the same Go/Nogo conflict task as in a previous study of our research group (Pscherer et al., 2019) to assess motor inhibitory control combined with an interference aspect. The task was administered with the software “Presentation” (Neurobehavioral Systems). It consisted of visual and auditory stimuli. As a visual stimulus, either the German word for “press” (“DRÜCK”) or the German word for “stop” (“STOPP”) was presented for 400 ms in white letters on a black background (21‐inch TFT screen).

Additionally, each visual stimulus was accompanied by an auditory stimulus presented via headphones. The auditory stimulus was also either the German word for “press” or the German word for “stop.” To provide emotional neutrality, those stimuli were created with “Google translate.” The participants were instructed to react to the visual stimuli exclusively and ignore the auditory ones. They were asked to press a button with their right index finger as quickly as possible when the visual stimulus “press” appeared, regardless of the auditory stimulus (Go trials). Furthermore, participants were asked to withhold their response when the visual stimulus “stop” appeared (Nogo trials), ignoring the auditory stimulus. In 50% of the trials, visual and auditory stimuli contained the same information, that is, both stimuli prompted the participants to “press” the button or to “stop” (= withhold) the response. Those cases were classified as “compatible” trials. The other 50% of trials were classified as “incompatible,” meaning that visual and auditory stimuli contained contradictory information (e.g., visual stimulus: “press” and auditory stimulus: “stop,” or vice versa). This leads to four different trial types: Go compatible, Go incompatible, Nogo compatible, and Nogo incompatible. Previous results have shown that in incompatible Nogo trials, response inhibition is stronger affected than in compatible Nogo trials (Chmielewski et al., 2016; Pscherer et al., 2019).

In total, 480 trials (336 Go trials, 144 Nogo trials) were presented in four blocks. The different trial types were distributed equally across the four blocks. With this 70:30 ratio of Go versus Nogo trials, the probability of premature responses (i.e., false alarms) was increased (Dippel et al., 2016; Young et al., 2018). Inter‐trial intervals were jittered between 1700 and 2100 ms. Go trials were coded as hits when a response was executed between 200 and 1200 ms after stimulus onset. Nogo trials were classified as correctly rejected if no response followed the stimulus within 1500 ms. If a response occurred in this time window, the trial was coded as a false alarm. Each Go trial ended after 1200 ms (even if the response was executed earlier), and each Nogo trial ended after 1500 ms (even if a response occurred). For the statistical analysis, the mean hit rate (given in percent), the mean reaction time on hits (given in ms), and the mean false alarm rate (given in percent) of each participant were computed.

### 2.3 EEG recording and analysis

The participants' EEG was recorded with BrainVision Recorder 2.1 (Brain Products Inc.) during the Go/Nogo conflict task performance. For this purpose, we used 60 Ag/AgCl electrodes in equidistant positions. The ground electrode was placed at position θ = 58, ϕ = 78, and the reference electrode at position θ = 90, ϕ = 90. We kept the electrode impedance below 5 kΩ. The EEG data were pre‐processed offline with BrainVision Analyzer 2.1 (Brain Products Inc.). First, the data was down‐sampled from the original sampling rate of 500 to 256 Hz. Then, we applied an IRR bandpass filter from 0.5 to 20 Hz (order 8) and a notch filter of 50 Hz. After that, we re‐referenced the data to the average activity of all channels and manually inspected the data to remove single technical and muscular artifacts. Subsequently, we carried out an independent component analysis (ICA, infomax algorithm) and thereby identified and manually removed repetitive artifacts, such as blinks, saccades, pulse, and muscular artifacts. The data was then imported into the MATLAB (R2020b, The MathWorks, MA, United States) software toolbox FieldTrip (Oostenveld et al., 2011). As a next step, the data was segmented based on the trial type (Go compatible vs. Go incompatible vs. Nogo compatible vs. Nogo incompatible) and locked to the stimulus onset. For the following steps and analyses, only correctly rejected Nogo trials were considered. The time windows of the segments reached from 2000 ms before to 2000 ms after stimulus onset. We applied an automated artifact rejection, which removed trials with an amplitude below −100 μV or above 100 μV or with activity below 0.5 μV in a time interval of 100 ms. A time‐frequency (TF) decomposition was computed on the single‐trial data of each participant and averaged over trials to analyze the total theta and alpha activity before and during the correctly rejected Nogo trials. For this purpose, we used Morlet wavelets with a Morlet parameter of 5 and computed the participants' average theta power (averaged between 4 and 7 Hz) as well as the average alpha power (between 8 and 12 Hz) for each channel and time point. No baseline normalization was performed. We compared the average within‐trial period (i.e., activity after stimulus onset averaged across trials per subject) between compatible and incompatible Nogo trials. Therefore, for both frequency bands, *t*‐tests were computed from 0 to 1000 ms relative to the stimulus onset for each time point and electrode across all participants. To correct for multiple comparisons, we applied the false discovery rate (FDR) method (Benjamini & Hochberg, 1995) to adjust *p*‐values. For further analysis of the within‐trial interval, a time window from 0 to 600 ms relative to stimulus onset was chosen, encompassing at least three complete theta frequency band cycles. This time window overlaps with the assumed time window of stimulus‐related cognitive processing as well as with the time intervals of significant differences. A time window of the same duration (600 ms) was chosen for the pre‐trial interval, ranging from −600 ms to target stimulus presentation.

### 2.4 Beamforming analysis

The study's goal was to investigate the role of theta and alpha activity in the relationship between proactive and reactive inhibitory control modulated by perceptual interference. Therefore, we planned to calculate correlations between pre‐trial and within‐trial theta and alpha activity at the source level. For this purpose, we reconstructed the source theta and alpha activity from the recorded scalp activity. A two‐step beamforming approach was applied first to identify relevant sources (i.e., neuroanatomical regions) of the measured pre‐trial and within‐trial activities and then to reconstruct the time courses of theta and alpha activity at these sources. This approach has also been applied in previous studies of our group (Adelhöfer & Beste, 2020; Dippel et al., 2017).

For the first step, dynamic imaging of coherent sources (DICS) beamformer was used to identify relevant clusters of voxels associated with pre‐ and within‐trial theta and alpha activity (Gross et al., 2001). This step was done separately for both frequency bands and both analysis windows. We performed a frequency transformation to calculate the cross‐spectral density matrix. Following the frequency band selection during the TF decomposition (see Section 2.3), theta power was averaged between 4 and 7 Hz to a central frequency of 5.5 Hz and a central frequency of 10 Hz for the alpha power frequency band (8–12 Hz). For the pre‐trial data and within‐trial data, common spatial filters were calculated over the covariance matrix of the combined Nogo trials of both congruency levels in the respective time frame. The EEG electrode positions were projected onto the forward model, which is included in the FieldTrip toolbox and based on the MNI template (see Oostenveld et al., 2003 for mathematical details). This template consists of grids with a resolution of 5 mm, and the leadfield matrix was computed for each grid point. Theta power was extracted for the pre‐trial and the within‐trial interval. The same was done for alpha power. We calculated the Neural Activity Index (NAI) for the pre‐trial windows by dividing the power values by the respective noise estimates for each voxel to account for the increased noise toward the center of the head (Van Veen et al., 1997). This was necessary since it was not possible to contrast any conditions in the pre‐trial interval. For the within‐trial interval, following the stimulus onset, we calculated a contrast between the Nogo incompatible and the Nogo compatible condition (Nogo incompatible − Nogo compatible) for each frequency band (theta and alpha) to examine the interference modulation effect of the inhibition/interference task. As no baseline window was available, the power difference was normalized on the sum of activity as $\text{ratio}=\left({\text{Nogo}}_{\text{incomp}}-{\text{Nogo}}_{\text{comp}}\right)/\left({\text{Nogo}}_{\text{incomp}}+{\text{Nogo}}_{\text{comp}}\right)$.

**FIGURE 1:**
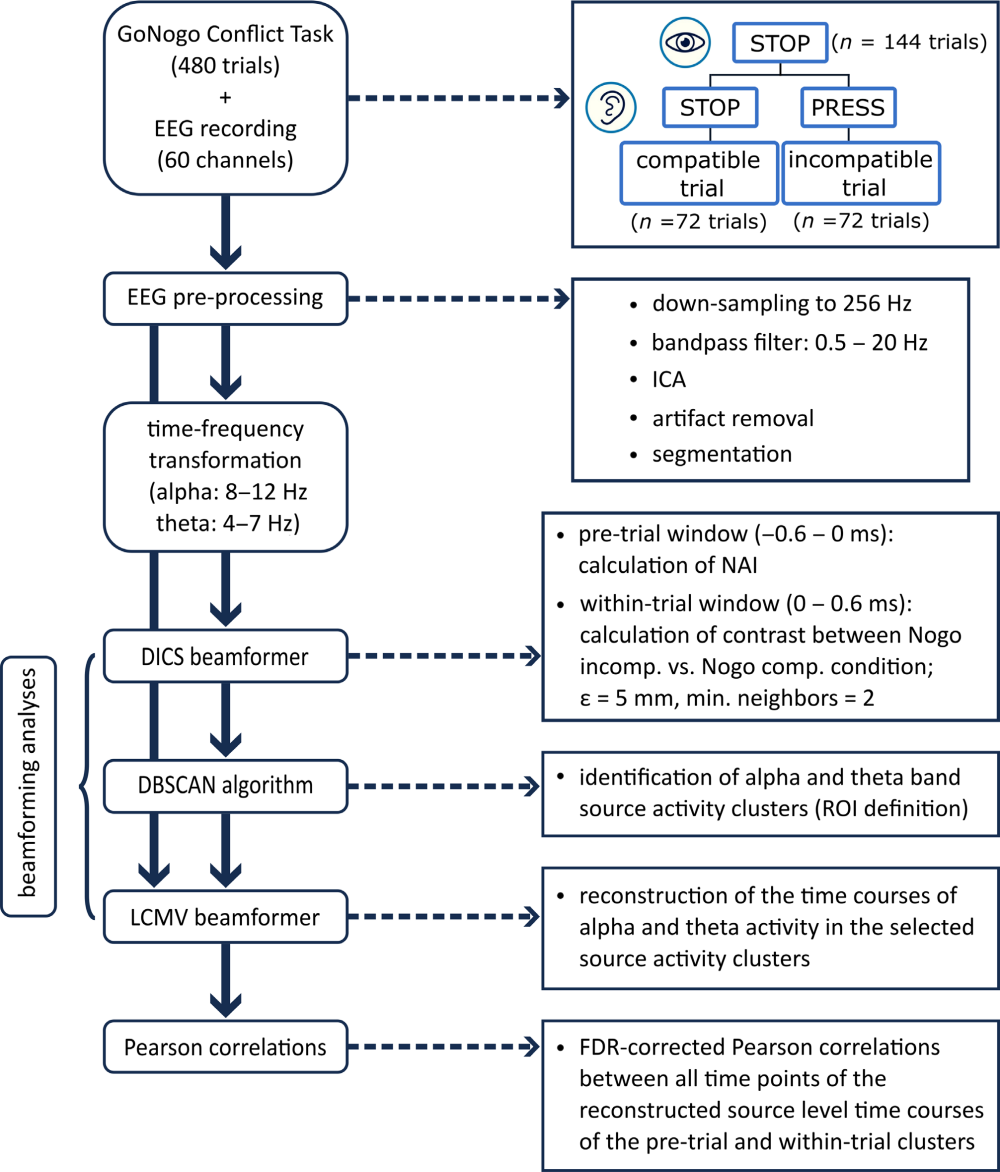
Analysis pipeline. On the left side, each step of the data analysis is depicted. The arrows with a continuous line represent the chronological sequence of the individual steps. The arrows with a dashed line lead to a brief explanation of the respective steps on the right side. Comp., compatible; DBSCAN, Density‐Based Spatial Clustering of Applications with Noise; DICS, Dynamic Imaging of Coherent Sources; FDR, False Discovery Rate; Hz, Hertz; ICA, Independent Component Analysis; incomp., incompatible; LCMV, Linear Constraint Minimum Variance; min. neighbors, minimum number of neighbors; NAI, Neural Activity Index; ROI, Region of Interest; ε, epsilon. Images of eye and ear: ©SvtDesign—Can Stock Photo Inc.

Subsequently, we applied a Density‐Based Spatial Clustering of Applications with Noise (DBSCAN) algorithm (Ester et al., 1996) to identify theta and alpha activity clusters in the DICS‐beamformed data. The DBSCAN algorithm identifies clusters by grouping data points that are located closely together without the need to specify an a priori number of clusters (Ester et al., 1996). Using this approach ensured that only voxels within functional neuroanatomical regions were included in further analyses (Adelhöfer & Beste, 2020; Adelhöfer et al., 2020). Since we wanted to limit the analysis to voxels with high theta power and high alpha power differences between the two conditions, respectively, we set the threshold of the power values to the top 2% of the power distribution within gray matter regions labeled in the Automatic Anatomical Labeling atlas (AAL) (Tzourio‐Mazoyer et al., 2002), excluding cerebellar structures. Epsilon (ε) was set to be equal to the edge length, that is, *ε* = 5 mm. The minimum number of neighbors was set to two. The clusters identified by the DBSCAN algorithm were visually inspected. Based on cluster size (minimum of 5 voxels per cluster) and neuroanatomical region (cerebellar regions and white matter structures were excluded), only the most important clusters were selected for subsequent analysis steps. Large clusters consisting of very different neuroanatomical regions were divided into separate clusters because the DBSCAN algorithm does not take into account the subdivision of the brain into neuroanatomical structures. For example, if the DBSCAN algorithm identified a cluster consisting of voxels in the insula, the inferior frontal gyrus, and the temporal pole, it was manually divided into three clusters according to the distinct neuroanatomical regions. That is, one cluster for the inferior frontal gyrus, one cluster for the insula, and one cluster for the temporal pole. To reconstruct the time course of theta and alpha activity in the selected source activity clusters, we performed a Linear Constraint Minimum Variance (LCMV) beamformer (Van Veen et al., 1997). Based on the covariance matrix of the averaged data in each condition, we computed the spatial filter of the LCMV beamformer for each cluster and multiplied it with the pre‐processed data. The source‐reconstructed time‐series were averaged across all voxels within each cluster. Next, time‐frequency representations within each cluster were computed for each participant with Morlet wavelets (same parameters as in the electrode‐level time‐frequency analysis) and averaged across trials. Subsequently, the power time courses of theta and alpha activity were extracted for every determined functional neuroanatomical region. The difference between theta power in the incompatible and the compatible Nogo condition was computed for each subject to reveal the interference effect during motor inhibition. The same was done for alpha power. As a final step, we calculated Pearson correlations across all subjects (*N* = 50) between every time point of the reconstructed time course at the source level of the pre‐trial clusters and every time point of the reconstructed time course at the source level of the within‐trial clusters resulting in a correlation matrix. This was done between the frequency bands to investigate the interrelation of theta and alpha band activity. To account for multiple comparisons, we used the FDR method (Benjamini & Hochberg, 1995), resulting in *q*‐values, which are *p*‐values that were adjusted for the FDR. Only *q*‐values smaller than .05 were considered significant. Figure 1 provides an overview of the analysis pipeline and the individual analysis steps.

### 2.5 Experimental design and statistical analysis

Our study was designed as a correlational study examining the relationship between the variables pre‐trial alpha activity, pre‐trial theta activity, within‐trial alpha activity during Nogo trials (*incompatible‐compatible*), and within‐trial theta activity during Nogo trials (*incompatible‐compatible*). *Incompatible‐compatible* refers to the effect of the perceptual conflict, which is analyzed by calculating the activation difference between the Nogo incompatible and the Nogo compatible condition. In general, correlations were only calculated across frequency bands within one timeframe (pre‐trial, within‐trial), for example, a pre‐trial theta cluster was correlated with a pre‐trial alpha cluster, but not with another pre‐trial theta cluster. However, for the within‐trial correlations, we additionally exploratively analyzed correlations between different clusters in the theta band. Details are explained in Section 3.3.1. We analyzed the descriptive behavioral data with IBM SPSS Statistics v26 using two‐tailed paired *t*‐tests. Mean values and standard deviation of the mean values are given. As an effect size, Cohen's *d* is reported. The correlation analyses conducted to analyze the described relationship between pre‐trial source activity (theta and alpha) and the within‐trial perceptual interference effect on inhibition (theta and alpha source activity during Nogo incompatible vs. Nogo compatible trials) across subjects, were done with a build‐in MATLAB (R2020b, The MathWorks, MA, United States) function embedded in custom scripts.

## 3 RESULTS

### 3.1 Behavioral data

The analysis of the behavioral data showed that the participants' reaction time during incompatible Go trials (352.57 ms ± 55.96) was significantly slower than during compatible Go trials (338.54 ms ± 53.34; *t*(49) = −7.82, *p* < .001, *d* = −1.11). The hit rate did not differ significantly between incompatible Go trials (98.84% ± 3.15) and compatible Go trials (99.00% ± 2.63; *t*(49) = 1.09, *p* = .281, *d* = 0.15). During Nogo trials, a conflict effect was evident in the sense that the false alarm rate was significantly higher for incompatible (26.46% ± 16.55) than for compatible trials (23.42% ± 17.77; *t*(49) = −2.56, *p* = .014, *d* = −.36).

### 3.2 Neurophysiological data: Theta and alpha power analysis

**FIGURE 2:**
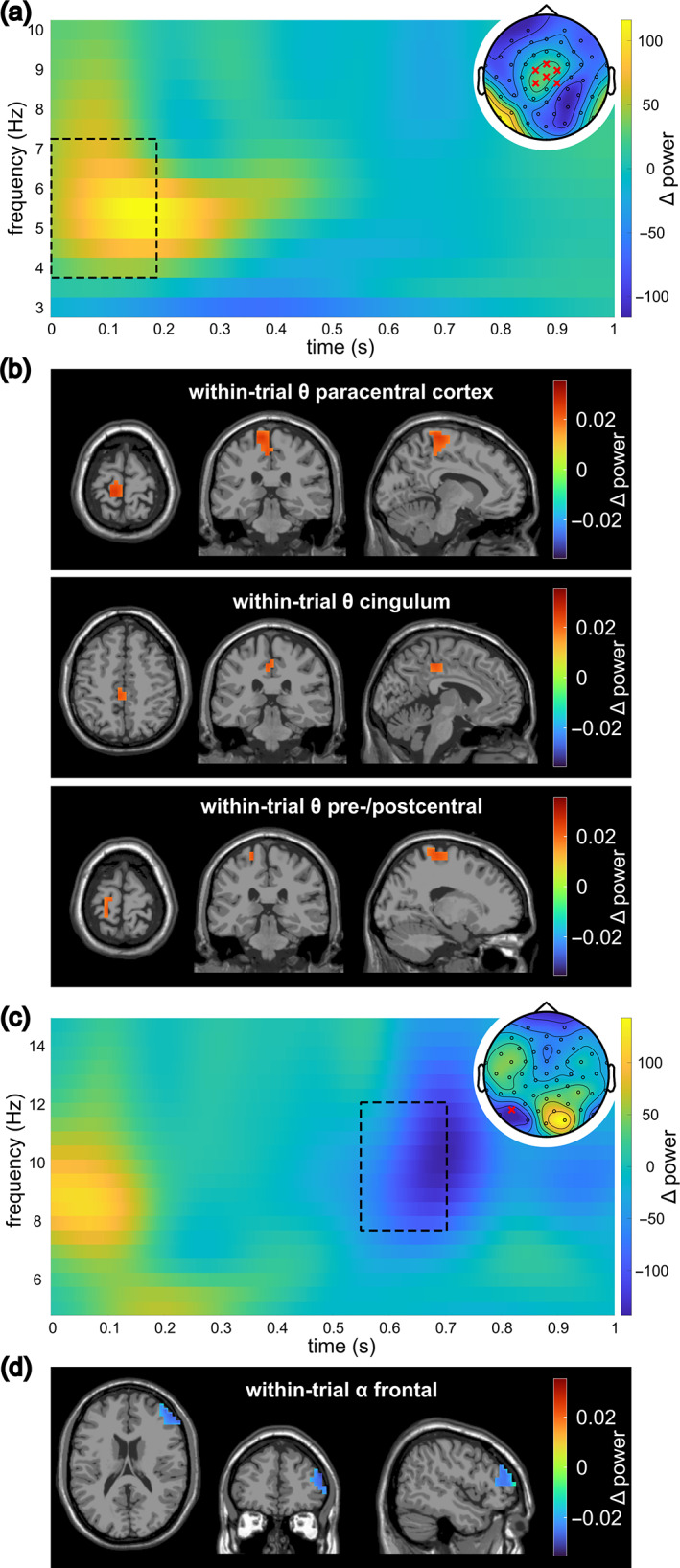
Summary of within‐trial results. (a) Time‐frequency representation over the significant fronto‐central electrodes (see topographic plot in the top right corner). Values represent the power difference of the incompatible − compatible condition. The square represents the time frame where the difference between incompatible and compatible Nogo trials was significant in FDR‐corrected *t*‐tests. The topographic plot shows the power difference in the theta band in the significant timeframe (0 to ~200 ms). The red crosses mark electrodes with significant power differences. (b) Within‐trial theta clusters of the conflict effect, as identified by beamforming and the DBSCAN algorithm (see Section 2) on the theta power difference between the incompatible and compatible Nogo condition. The color of the cluster scales the power difference between the incompatible and compatible Nogo condition. (c) Time‐frequency representation over electrode P7 (see topographic plot). Values represent the power difference between the incompatible and compatible Nogo condition. The square indicates the area of significant differences between the two conditions in the alpha band; the topographic plot shows the distribution of alpha power differences in the significant timeframe (~550 to 700 ms). (d) The within‐trial alpha cluster associated with the conflict effect as defined by beamforming and subsequent clustering with the DBSCAN algorithm. The color of the cluster scales the power difference between the incompatible and compatible Nogo condition.

We computed FDR‐corrected *t*‐tests to compare the subjects' average theta power in incompatible versus compatible Nogo trials in the within‐trial interval. The results revealed that from stimulus onset to 200 ms after stimulus onset theta power was significantly higher during the incompatible condition at central and frontocentral channel positions (Cz, FCz, FC1, CP1, FC2, CP2), as well as at temporal channel positions (TP9, P11, TP10, P12). In a later time window (450–700 ms after stimulus onset), theta power was higher in the compatible condition, but only at electrode P11. Figure 2a depicts the time‐frequency representation of the compatibility effect at the fronto‐central channels in the theta power band and the corresponding topographic plot.

On the source level, the DBSCAN algorithm identified three clusters of theta‐band activity contrasting the incompatible with the compatible Nogo condition. One cluster was found in the left hemispheric paracentral lobule (44 voxels), one in the left midcingulate cortex (11 voxels), while the last cluster consisted of voxels in the left‐hemispheric pre‐ and post‐central gyrus (13 voxels; see Figure 2b). For alpha activity in the within‐trial interval, the FDR‐corrected t‐tests showed significantly higher activity during compatible than during incompatible Nogo trials across subjects at electrode P7 from 550 to 700 ms after stimulus onset (see Figure 2c). The DBSCAN algorithm revealed one cluster in the middle and inferior frontal cortex (44 voxels; see Figure 2d). In the supplemental material, the time‐frequency data of each within‐trial cluster after LCMV beamforming are visualized (Figure S1).

**FIGURE 3:**
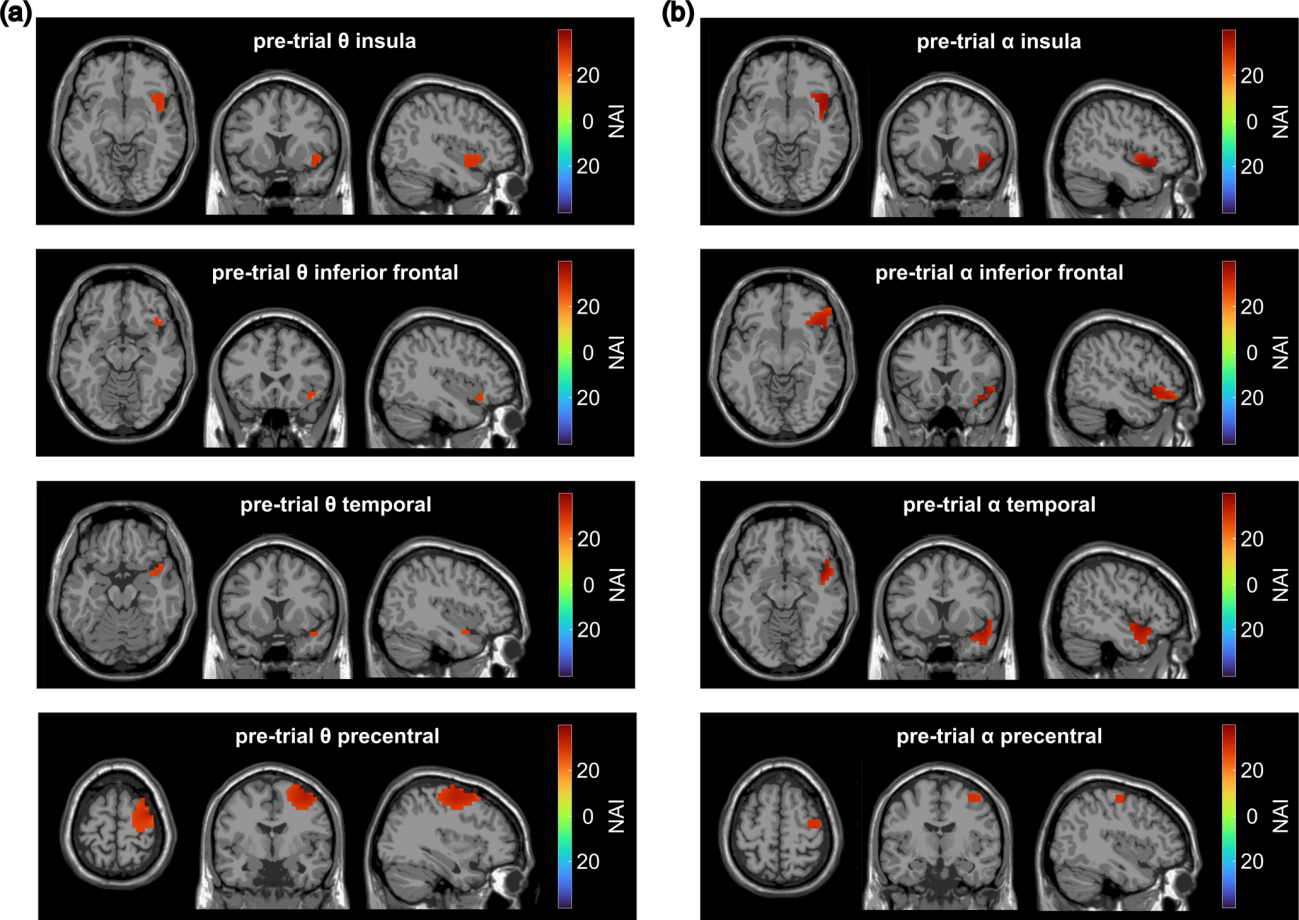
Summary of the pre‐trial clusters. (a) The four clusters of theta band activity in the pre‐trial period as identified by beamforming and subsequent application of the DBSCAN algorithm. (b) The four alpha clusters identified for the pre‐trial period. The color of the clusters in both parts of the figure represents the magnitude of the Neural Activity Index (NAI).

Subsequently, clusters were identified for the pre‐trial interval. Since this time period did not contain any cues on the following trial type, we could not calculate a contrast between incompatible and compatible Nogo pre‐trial intervals. Therefore, it could not be tested statistically whether pre‐trial activity (in the theta and alpha band, respectively) differed significantly between conditions or from a random signal. Usually, such a contrast statistically justifies the beamforming analysis. Yet, the analyses are based on correlations between reconstructed time courses in the identified source clusters. Since the LCMV beamformer (for the reconstruction of the time courses on the source level) is applied to the original signal, we can assume that a random signal in the pre‐trial interval would not lead to significant correlations after the FDR‐correction. Thus, the FDR‐corrected correlation analyses validate the source clusters in the pre‐trial interval identified by the DBSCAN algorithm (Wendiggensen et al., 2022). For pre‐trial theta activity, one cluster in the insula (23 voxels), one cluster in the inferior frontal gyrus (7 voxels), one cluster in the temporal pole (6 voxels), and one cluster comprised of the precentral and middle and superior frontal cortex (153 voxels) were found. All clusters were located in the right hemisphere. For pre‐trial alpha activity, four similar clusters were detected: one cluster in the right‐hemispheric insula (47 voxels), one in the right‐hemispheric inferior frontal cortex (54 voxels), one in the right‐hemispheric temporal pole (49 voxels) and one cluster in the right‐hemispheric precentral gyrus (11 voxels). Pre‐trial activity clusters are depicted in Figure 3. In the supplemental material, the time‐frequency data of each pre‐trial cluster after LCMV beamforming are visualized (Figure S2). Additionally, a supplemental table provides an overview of all DBSCAN clusters and their AAL regions (Table S1).

### 3.3 Neurophysiological data: Correlation analysis

We correlated the reconstructed time courses at the source level of all identified clusters with each other. Please note that only significant correlations after FDR correction are reported. The correlation matrices in the within‐trial interval were calculated for the conflict effect during motor inhibition. This means that for instance the time course of the power difference between incompatible and compatible Nogo trials in one cluster was correlated with the time course of the power difference in another cluster across all subjects.

#### 3.3.1 Within‐trial correlations (reactive control processes)

**FIGURE 4:**
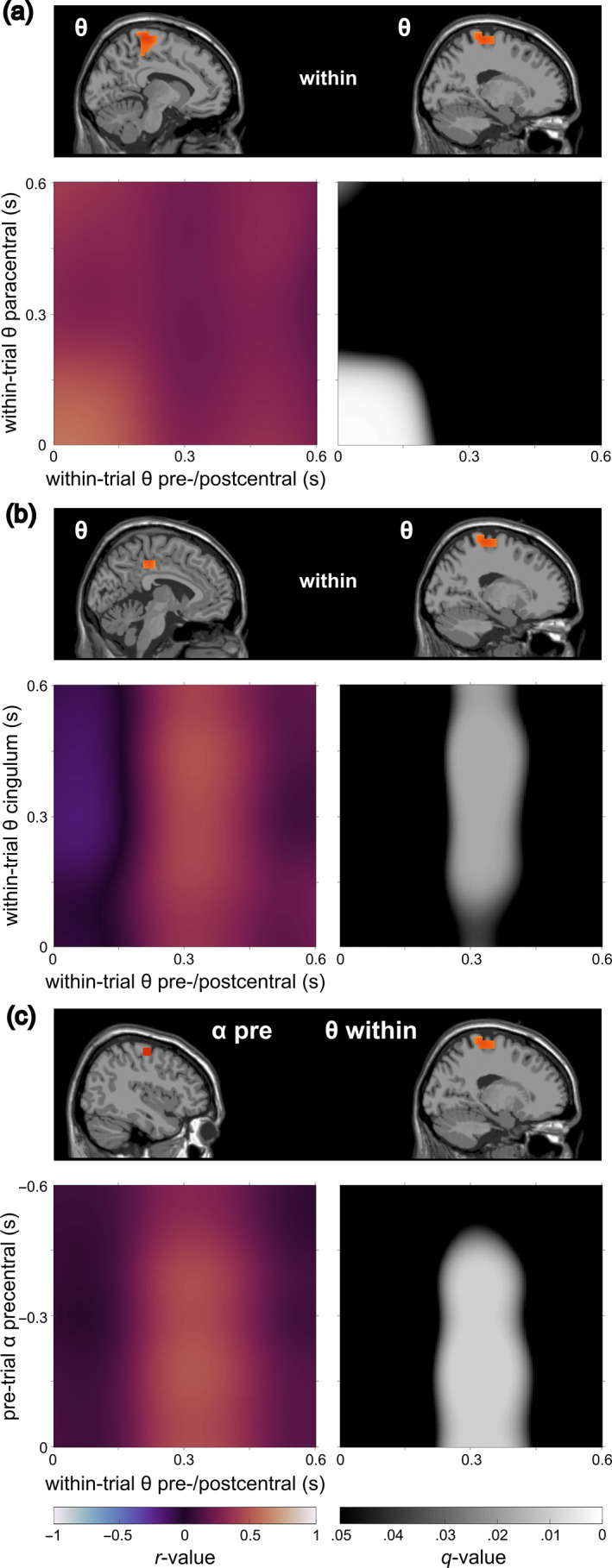
Summary of the main findings of the correlations in the within‐trial interval (0 to 0.6 s) after stimulus onset (a and b) and between the pre‐ and within‐trial interval (c). Please note that only significant correlation patterns are displayed. In each section, the top plot visualizes the location of the correlated clusters. Below, the correlation is depicted via a correlation matrix of the activity time courses within the respective clusters. The left plot scales the magnitude of the correlation (*r*‐value, indicated by color; see color bar at the bottom left of the figure). The right plot visualizes FDR‐corrected *q*‐values (indicated by color; see color bar at the bottom right of the figure). Black areas indicate no significance (*q* > .05), while lighter areas visualize significant correlations (*q* < .05).

The within‐trial correlations showed no significant associations between the conflict effect in the theta band and the conflict effect in the alpha band. The aim of the study was to better understand the neurophysiological dynamics underlying response inhibition modulated by a perceptual conflict. Since not only the interaction between different frequency bands but also the exact dynamics within the individual frequency bands are still not fully understood, we additionally performed exploratory correlation analyses between the different within‐trial clusters in the theta band. In the alpha band, only one cluster was evident, so that no further correlations could be calculated. The exploratory analysis revealed that the conflict effect in the paracentral theta cluster was positively correlated with the conflict effect in the pre‐ and postcentral theta cluster in the time frame from 0 to ~200 ms (*r*_max_ = 0.60, *r*_min_ = 0.38, within *q* < .05), indicating that a larger conflict effect in the paracentral theta cluster was related to a larger conflict effect in the pre‐ and postcentral theta cluster. The results further revealed that the conflict effect in the pre‐ and postcentral theta cluster in the time interval from about 250–400 ms after stimulus onset was positively correlated with the conflict effect in the midcingulate theta cluster from stimulus onset until the end of the trial (*r*_max_ = 0.49, *r*_min_ = 0.35, within *q* < .05). A higher conflict effect in the respective midcingulate theta time window was related to a higher conflict effect in the respective pre‐ and postcentral theta time window or vice versa. The correlation matrices for the within‐trial are shown in Figure 4a,b.

#### 3.3.2 Pre‐trial correlations (proactive control processes)

**FIGURE 5:**
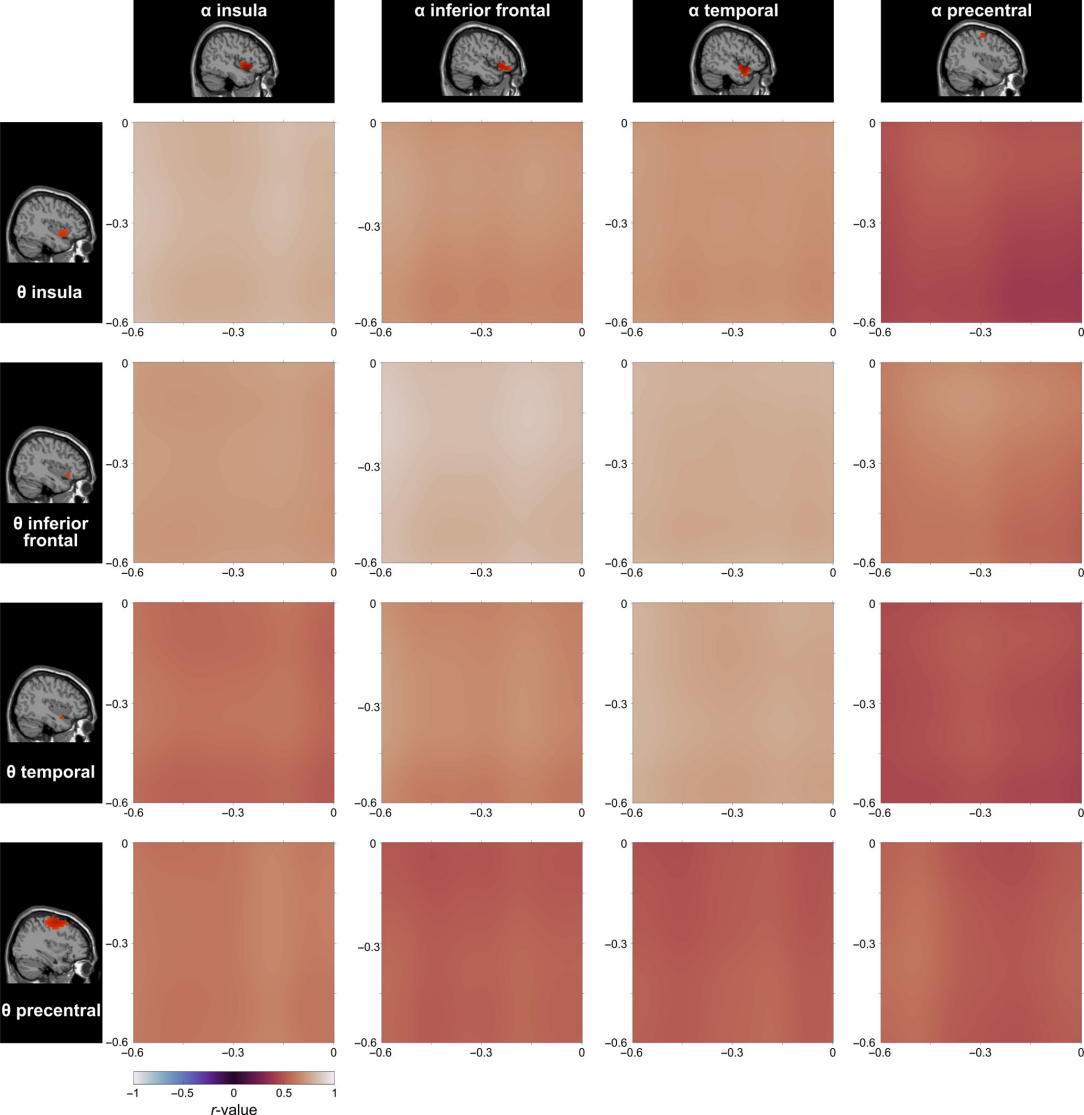
Summary of the correlations in the pre‐trial interval. The four pre‐trial theta clusters are depicted on the left side while the four pre‐trial alpha clusters are depicted at the top. Each correlation plot shows the correlation between the clusters on the respective locations at the axes. The correlation plot visualizes the magnitude of the correlation coefficient between the two respective time points (*r*‐value, indicated by color; see color bar at the bottom left of the figure).

The correlation maps in the pre‐trial time interval revealed significant positive correlations (*q* < .05) between all pre‐trial theta cluster time courses and the pre‐trial alpha cluster time courses throughout the entire time window (−600 ms to stimulus onset). The correlation between the pre‐trial insular alpha cluster and the pre‐trial insular theta cluster ranged from *r*_min_ = 0.77 to *r*_max_ = 0.85. Slightly lower correlation coefficients were found for the correlations between the insular theta cluster and the inferior frontal alpha cluster (*r*_min_ = 0.65, *r*_max_ = 0.76) and between the insular theta cluster and the temporal alpha cluster (*r*_min_ = 0.67, *r*_max_ = 0.73). The correlation coefficients ranged from *r*_min_ = 0.39 to *r*_max_ = 0.55 between the insular theta cluster and the precentral alpha cluster. The pre‐trial inferior frontal theta cluster showed significant positive correlations with the insular alpha cluster (*r*_min_ = 0.70, *r*_max_ = 0.75), the inferior frontal alpha cluster (*r*_min_ = 0.78, *r*_max_ = 0.89), the temporal alpha cluster (*r*_min_ = 0.76, *r*_max_ = 0.82) and the precentral alpha cluster (*r*_min_ = 0.53, *r*_max_ = 0.71). The pre‐trial temporal theta correlated significantly with the insular alpha cluster (*r*_min_ = 0.51, *r*_max_ = 0.63), the inferior frontal alpha cluster (*r*_min_ = 0.61, *r*_max_ = 0.74), the temporal alpha cluster (*r*_min_ = 0.74, *r*_max_ = 0.81) and the precentral alpha cluster (*r*_min_ = 0.43, *r*_max_ = 0.53). The correlations between the precentral theta cluster and the alpha clusters ranged from 0.47 to 0.66 (insular alpha cluster: *r*_min_ = 0.59, *r*_max_ = 0.66; inferior frontal alpha cluster: *r*_min_ = 0.48, *r*_max_ = 0.57; temporal alpha cluster: *r*_min_ = 0.47, *r*_max_ = 0.57; precentral alpha cluster: *r*_min_ = 0.47, *r*_max_ = 0.61). The correlation matrices of the pre‐trial time window are shown in Figure 5. Table S2 contains the mean *R* ^2^ values for each pre‐trial correlation matrix.

#### 3.3.3 Pre‐trial/within‐trial correlations (interrelation between proactive and reactive control processes)

The analysis of pre‐trial/within‐trial activity correlations showed a significantly positive correlation (*r*_max_ = 0.54, *r*_min_ = 0.34, within *q* < .05) between pre‐trial alpha activity in the precentral cluster from −500 ms to stimulus onset and the within‐trial theta conflict effect in the pre‐/postcentral cluster from ~230 to ~400 ms after stimulus onset. In other words, a higher pre‐trial alpha activity was related to a higher within‐trial theta conflict effect (or vice versa) in the respective brain regions and time intervals. The corresponding correlation matrix is depicted in Figure 4c.

## 4 DISCUSSION

**FIGURE 6:**
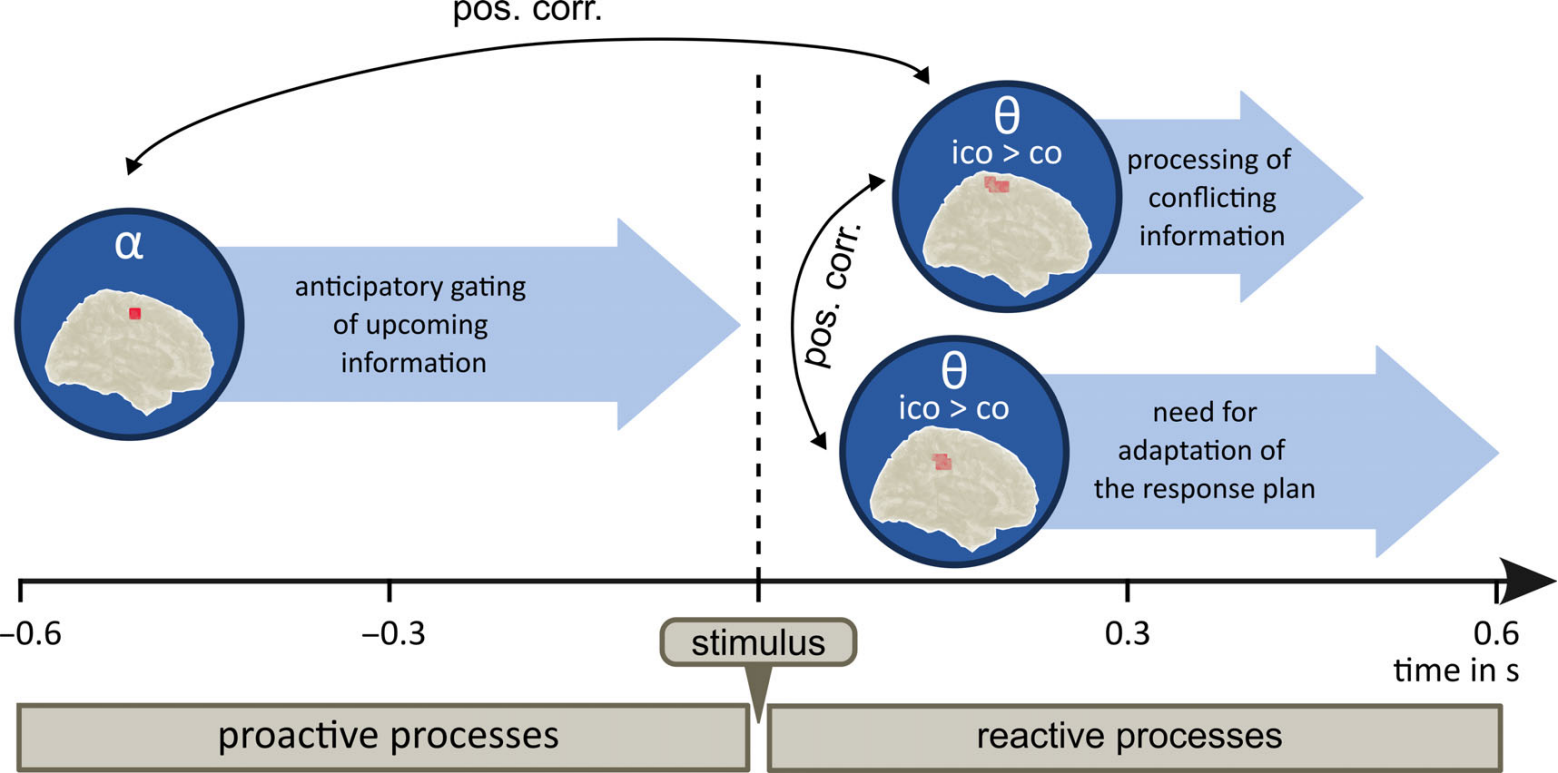
Schematic illustration of the relationships between proactive (pre‐trial) and reactive (within‐trial) processes during conflict‐modulated motor inhibition. Pre‐trial alpha activity in the primary motor cortex is positively correlated with the within‐trial theta conflict effect in the primary motor cortex (~300 ms after stimulus onset). “Ico > co” refers to the fact that theta power was higher in incompatible Nogo trials than in compatible Nogo trials. This within‐trial theta conflict effect in the primary motor cortex is then positively correlated with the within‐trial theta conflict effect in the midcingulate cortex until the end of the trial. These interrelations suggest that participants with stronger anticipatory information gating in the primary motor cortex (alpha band) show stronger reactive processing of the perceptual conflict in the primary motor cortex (theta band). The results further imply that as a next step, participants with stronger processing of the perceptual conflict in the primary motor cortex (theta band) show a stronger realization of the need for adapting the current response plan in the midcingulate cortex (theta band). In sum, theta and alpha band activity seem to share and transfer aspects of information when it comes to the interrelationship of proactive and reactive control during conflict‐modulated motor inhibition.

In the current study, we examined the neurophysiological dynamics underlying perceptual conflict modulations of inhibitory control with a focus on theta and alpha band activity. For a particularly comprehensive approach, we examined the relevance of both frequency bands within the trials (reactive control processes) and additionally considered neurophysiological processes before trial onset (i.e., pre‐trial activity) to engage in proactive cognitive control. Importantly, pre‐ and within‐trial activities were correlated (see below for details) to examine the interplay of proactive control and cognitive control during response inhibition. The behavioral data replicate previous results (Chmielewski et al., 2016; Pscherer et al., 2019) and show that response inhibition performance, as measured by false alarms, worsens when perceptual input is conflicting. Below, we first discuss the reactive control dynamics before going into the proactive control processes, as well as the interrelation between proactive and reactive control aspects. Figure 6 provides a schematic illustration of the revealed relationships between proactive (pre‐trial) and reactive (within‐trial) processes during conflict‐modulated motor inhibition.

### 4.1 Reactive control processes

Conflict effects were associated with an increase in theta band activity during incompatible compared to compatible Nogo trials. This effect is reasonable: Medial and superior frontal theta band activity has repeatedly been shown to be elevated during conflicting information (Cohen, 2014; Hoffmann et al., 2014; Nigbur et al., 2011; Pscherer et al., 2020), possibly reflecting the need to increase cognitive control capacities to maintain task performance (Cavanagh & Frank, 2014). Wendiggensen et al. (2022), however, found opposing effects in a Simon Nogo task with higher theta power during congruent than incongruent Nogo stimuli. However, this does not contradict the current findings, because the source of conflicts in a Simon tasks is a stimulus–response conflict (Hommel, 2011; Spapé et al., 2011; Wiegand & Wascher, 2007) and not a perceptual one as in the current study. The beamforming analysis of our data showed that theta activity modulations were related to paracentral, precentral, and postcentral regions, as well as to the midcingulate cortex in the left hemisphere. The para‐, pre‐, and postcentral cluster overlaps with the primary motor cortex, which fits to a previous finding that theta power is elevated especially in central regions during motor inhibition (Nguyen et al., 2021). The primary motor cortex is involved in action planning and selection, especially when an existing action plan is challenged by incoming stimulus information (Calderon et al., 2018; Pape & Siegel, 2016). Our findings suggest that particularly the effect of a perceptual conflict on motor‐related processes during inhibitory control is reflected by modulations in central theta band activity (see Figure 2b).

Activity modulations observed in the midcingulate cortex may also be interpreted along these lines since the midcingulate cortex contains (cingulate) premotor areas (Vogt, 2016). Furthermore, the anterior midcingulate cortex has been shown to be crucially involved in a functional network implementing the realization of intentional motor control (Hoffstaedter et al., 2013). Interestingly, parietal afferents terminate in the midcingulate cortex (Vogt, 2016). Since the parietal cortex has been related to perceptual processes (Bodmer & Beste, 2017; Santangelo et al., 2015) and input integration (Gottlieb, 2007; Gottlieb & Snyder, 2010; Pscherer et al., 2022), the activation in the midcingulate cortex could also reflect theta band‐related perception‐action integration processes. The suggested functional role of perception‐action integration is underpinned by the finding that dynamic representations of goal‐directed actions are encoded in the midcingulate cortex (Holroyd et al., 2018; Vogt, 2016). Hence, an increased theta activity in the midcingulate cortex might indicate a challenged mental representation of the action plan due to conflicting stimulus information and thus a need for adapting the response plan.

Contrary to our expectations, alpha band activity was decreased in incompatible compared to compatible Nogo trials. While other studies showed that alpha activity in the lateral occipital (Wiesman & Wilson, 2020) and posterior parietal cortex (Jiang et al., 2018) was related to interference conflicts, in the current study the conflict‐related activation differences were located in the right middle and inferior frontal gyrus and occurred in a rather late time window (550 to 700 ms after stimulus onset). The right inferior frontal gyrus has been shown to be involved in inhibitory control processes and attentional monitoring (Aron, 2011; Bari & Robbins, 2013). Alpha band activity likely mediates processes related to the suppression of information in task‐irrelevant networks and thereby controls the access to relevant information for behavior (Klimesch, 2011, 2012). Since Nogo trials with a perceptual conflict require the inhibition of irrelevant stimulus information, we had expected a higher alpha band activity in Nogo trials with versus without a perceptual conflict. However, two recent studies found similar results to ours in a Go/Nogo task contrasting a more complex Nogo condition (overlapping features with Go trials) with a less complex Nogo condition (no overlapping features with Go trials) (Prochnow, Eggert, et al., 2022; Prochnow, Wendiggensen, et al., 2022). In these studies, alpha band activity in frontoparietal and occipital regions including the right inferior frontal gyrus was decreased in the more complex versus the less complex Nogo condition. The authors concluded that a “more straightforward inhibitory gating of information”—reflected by comparably increased alpha band activity—was possible when the stimulus did not contain conflicting perceptual information (Prochnow, Eggert, et al., 2022). The authors' findings are in line with our results suggesting that ambiguous stimulus information impedes successful inhibitory gating and thus allows to process irrelevant information, which is reflected by decreased alpha band activity. This could explain the higher false alarm rate during Nogo trials with versus without a perceptual conflict.

The correlation analyses revealed that reactive control‐related alpha and theta band dynamics did not correlate. Interestingly, however, the conflict effect in the theta band in the pre‐ and postcentral cluster, that is, in the primary motor cortex (around 300 ms after stimulus onset), correlated positively with the conflict effect in the theta band in the midcingulate cortex (0–600 ms after stimulus onset). The finding suggests that participants with a higher theta‐related conflict effect (i.e., increased theta band activity in incompatible vs. compatible Nogo trials) in the primary motor cortex around the average reaction time showed a higher conflict effect in the cingulum until 600 ms after stimulus onset. On a functional level, this correlational pattern might imply that the realization of a need to adapt the current representation of an action plan is transferred between the primary motor cortex and the midcingulate cortex via theta band activity. According to the temporal pattern of the correlation, participants with a stronger processing of the perceptual conflict in the primary motor cortex around 300 ms after stimulus onset show a stronger realization of the need for adapting the representational action plan in the midcingulate cortex until (at least) 600 ms after stimulus onset. The theta band‐related processes in the primary motor cortex thus might have a relatively “long”‐lasting impact on the theta band‐related processes in the midcingulate cortex. A second significantly positive correlation between theta clusters was shown in a very early time window (0–200 ms after stimulus onset): Participants with a stronger conflict effect in the theta band in the paracentral region showed a stronger theta‐related conflict effect in the pre‐ and postcentral cluster. Since this effect was evident in a very early time window, it is difficult to explain and lacks a meaningful interpretation.

### 4.2 Proactive control processes and interrelation with reactive control processes

The above discussion suggests that reactive conflict effects in the alpha band in the right middle and inferior frontal gyrus represent differences in inhibitory gating. Proactive (pre‐trial) control processes in the alpha band, however, were observable not only in the orbital part of the right inferior frontal gyrus but also in the right insula, the right superior temporal pole, as well as in the right precentral gyrus. Contrary to our expectations, we found no proactive alpha activity in the parietal cortex and superior frontal cortex. We assume that the observed pre‐trial processes reflect proactive control processes since it has previously been suggested that proactive control processes may be evident in the pre‐trial interval (Adelhöfer & Beste, 2020; Adelhöfer et al., 2021). Interestingly, in almost the same regions, proactive control processes were evident in the theta band. The correlation analyses revealed that alpha band activity and theta band activity in all pre‐trial clusters were significantly positively correlated. That is, for example, participants with a higher proactive alpha activity in the insula showed a higher proactive theta activity in the insula and all other theta clusters. We assume that the insula was involved due to our multisensory task design (visual and auditory stimuli) since previous findings suggest that the insula is a crucial brain region when it comes to integrating various sensory inputs and sensory‐motor integration (Gogolla, 2017; Perri et al., 2018).

Theta activity in the inferior frontal cortex and superior temporal gyrus has been related to attentional sampling processes in a task combining conflict and inhibition processes (Wendiggensen et al., 2022). A higher proactive theta activity was associated with a higher reactive theta band modulation between congruent and incongruent Nogo trials during response inhibition (Wendiggensen et al., 2022). While the interpretation that pre‐trial activity may reflect proactive control and attentional sampling has previously only been related to the theta band, the current data show that alpha and theta band activities in the pre‐trial interval were strongly positively correlated (see Figure 5). Both frequency bands are thus likely to reflect similar processes, which also supports the interpretation that the observed pre‐trial alpha‐band activity may reflect aspects of proactive control. This is also plausible considering the functional role of alpha‐band activity in the (anticipatory) gating of upcoming information (Foxe & Snyder, 2011; Klimesch, 2012).

To obtain a clearer idea of the processes involved in conflict‐modulated response inhibition, we correlated proactive and reactive control processes. The analysis revealed a significantly positive correlation between alpha band‐related proactive control in the precentral gyrus (i.e., the primary motor cortex) and theta band‐related reactive control in the pre‐ and postcentral gyrus. The results showed that participants with a higher proactive alpha control in the primary motor cortex from 500 ms before stimulus onset onwards show a higher reactive conflict effect in the theta band from 230 to 400 ms after stimulus onset in the same region. Research in primates has shown that in the primary motor cortex, anticipatory planning and preparation of a motor response takes place (Confais et al., 2012; Lu & Ashe, 2005). Together with the suggestion that alpha band activity plays a crucial role in anticipatory information gating (Foxe & Snyder, 2011; Klimesch, 2012), our results thus suggest the following: when anticipatory gating processes relevant for the preparation of the upcoming motor response are strong, the realization of the need to adapt the mental representation of the action plan is strong as well.

Taken together, the resulting pattern from the correlational analyses showing a particular temporal profile of the obtained correlations of theta/alpha band activity between brain regions might suggest a cascade of processes that is delineated in Figure 6 (see above): Pre‐trial alpha band activity in the primary motor cortex reflects anticipatory information gating which is necessary for preparing the upcoming motor response. Participants with strong proactive information gating in the primary motor cortex show a strong realization of the need to adapt the action plan in reaction to conflicting stimulus information during motor inhibition. This need to adjust the mental representation of the action plan is then transferred to the midcingulate cortex until the end of the trial. Alpha and theta band activity thus seem to share and maybe transfer information from the primary motor cortex to the cingulum when it comes to response inhibition modulated by a perceptual conflict. Participants with stronger proactive information gating seem to realize the perceptual conflict stronger and thus signal a need to adapt the action plan. This implies that brain activity states in the pre‐trial period contain important information for subsequent cognitive control processes. Therefore, pre‐trial activity should not only be considered as dispensable baseline activity but as a correlate of brain states affecting later cognitive processing (Beste et al., 2023). Interestingly, pre‐trial alpha activity seems to be especially relevant to prepare for perceptual conflicts during response inhibition, whereas pre‐trial theta activity seems to be of importance for proactive inhibitory control at the motor level (Wendiggensen et al., 2022).

Importantly, however, our correlations were calculated across subjects and not trial‐wise. Therefore, the results can only be interpreted based on subject‐related differences, suggesting, for example, that individuals with higher proactive control/anticipatory gating show a stronger perceptual conflict effect. Future research should consider analyzing trial‐wise dynamics to corroborate the current findings. By this means it could be analyzed whether single trials with higher proactive control are followed by lower reactive control activity. A caveat is that the correlation analyses do not allow for causal inferences. However, for investigating the interplay of activation changes over time, this method provides profound insights into the temporal sequence of the described processes. Future studies should take these aspects into account and extend the investigation to other domains of cognitive control and other frequency bands. Interregional relationships should further be investigated by coherence and phase synchrony analyses. Moreover, the measure of proactive control was not based on a cue stimulus signaling participants to prepare for the upcoming stimulus, which might limit the distinguishability between evaluation processes of the previous trial and preparational/proactive processes concerning the upcoming trial. To narrow down the pre‐trial processes more precisely, a replication of the study including cue stimuli signaling the onset of the upcoming trial would provide further insight into proactive control processes. Finally, there are pronounced hemispheric differences between the activities before (right hemispheric activity) and during (left hemispheric activity) the trial. The reasons for these differences are currently unclear and should be explored in future research.

## 5 CONCLUSION

In conclusion, our data showed that proactive and reactive theta and alpha activity are related to motor inhibition processes which are modulated by an auditory–visual perceptual conflict. Especially during (pre‐trial) proactive control, alpha activity in the right inferior frontal gyrus, the right insula, the right superior temporal pole, as well as in the right precentral gyrus was strongly positively correlated with theta activity in the same regions as well as in the right superior frontal gyrus. Participants with a stronger proactive control‐related alpha activity in the primary motor cortex further showed a stronger conflict effect in the theta band in the primary motor cortex, probably reflecting a stronger conflict processing due to successful proactive preparation, that is, anticipatory information gating. Additionally, participants with a stronger theta‐related conflict effect in the primary motor cortex showed a stronger theta‐related conflict effect in the midcingulate cortex, indicating a transfer of the need to adapt the current action plan. The results imply that theta and alpha band activity share and maybe transfer aspects of information when it comes to the interrelationship of proactive and reactive control during conflict‐modulated motor inhibition.

## AUTHOR CONTRIBUTIONS

All authors had full access to the data, gave final approval for publication and agree to be held accountable for the work performed therein. *Conceptualization*: Charlotte Pscherer, Christian Beste, Moritz Mückschel, and Annet Bluschke. *Software*: Paul Wendiggensen and Moritz Mückschel. *Investigation*: Charlotte Pscherer. *Formal analysis*: Charlotte Pscherer, Paul Wendiggensen, and Moritz Mückschel. *Writing – original draft*: Charlotte Pscherer, Paul Wendiggensen, and Christian Beste. *Writing – reviewing & editing*: Charlotte Pscherer, Paul Wendiggensen, Moritz Mückschel, Annet Bluschke, and Christian Beste. *Visualization*: Paul Wendiggensen and Charlotte Pscherer. *Supervision*: Christian Beste. *Funding acquisition*: Christian Beste.

## FUNDING INFORMATION

This work was partly supported by a Grant from the Deutsche Forschungsgemeinschaft (DFG) SFB940 project B8.

## CONFLICT OF INTEREST STATEMENT

The authors declare no conflicts of interest.

## Supporting information


**Data S1:** Supporting Information.

## Data Availability

The data that support the findings of this study are available from the corresponding author upon reasonable request.
