# Supplementary material for: Alpha and theta band activity share information relevant to proactive and reactive control during conflict‐modulated response inhibition
Source: Hum Brain Mapp. 2023 Sep 20;44(17):5936–52. doi: 10.1002/hbm.26486 (PMC10619371; doi:10.1002/hbm.26486)
Supplement: Supplementary file 1 — Data S1: Supporting Information. [file HBM-44-5936-s001.pdf]

Supplemental Material

Alpha and theta band activity share information relevant to proactive and reactive control during conflict-modulated response inhibition

Charlotte Pscherer, Paul Wendiggensen, Moritz Mückschel, Annet Bluschke, Christian Beste

Supplemental analysis 1

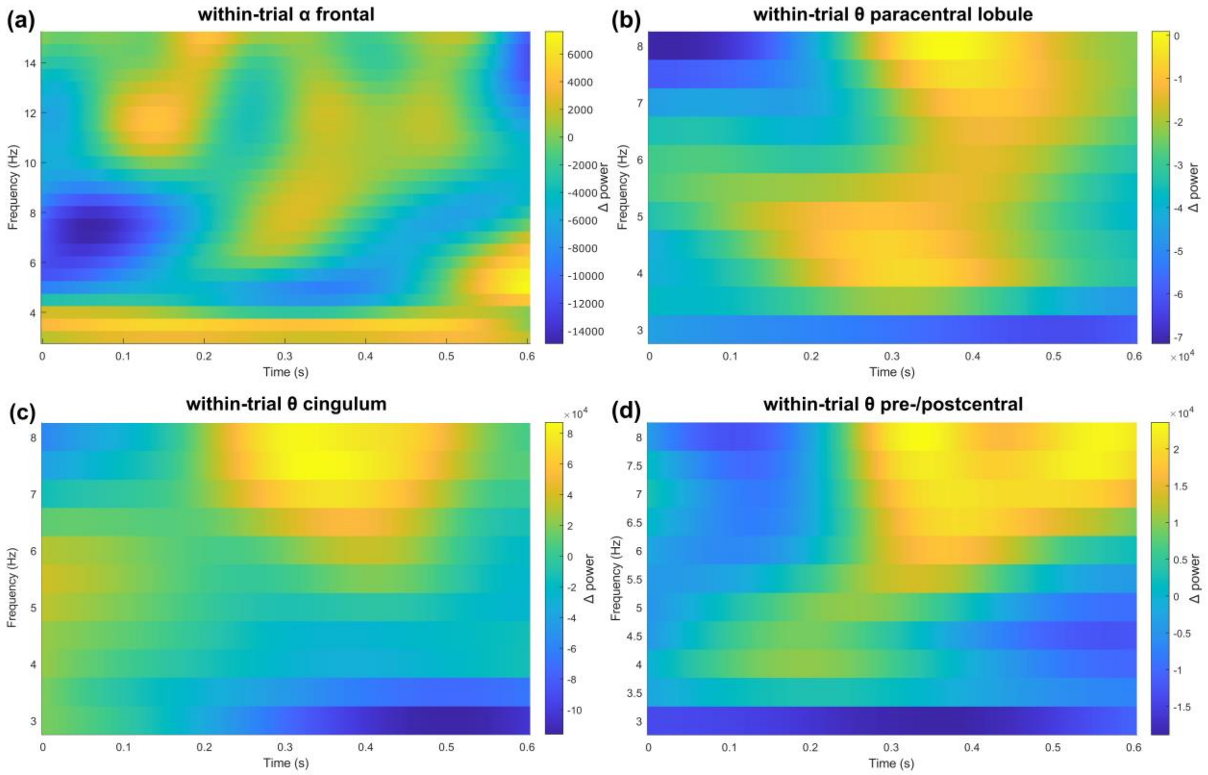

Supplemental figure 1.
Visualization of the time-frequency data after LCMV beamforming for each of the within-trial clusters. Figure parts **(a-d)** show the time-frequency representation for each within-trial cluster (0 to 0.6 s relative to stimulus presentation). The x-axis represents the time in the trial while the y-axis represents the frequencies. The color of the plot indicates the magnitude of the power difference between the incompatible and compatible condition.

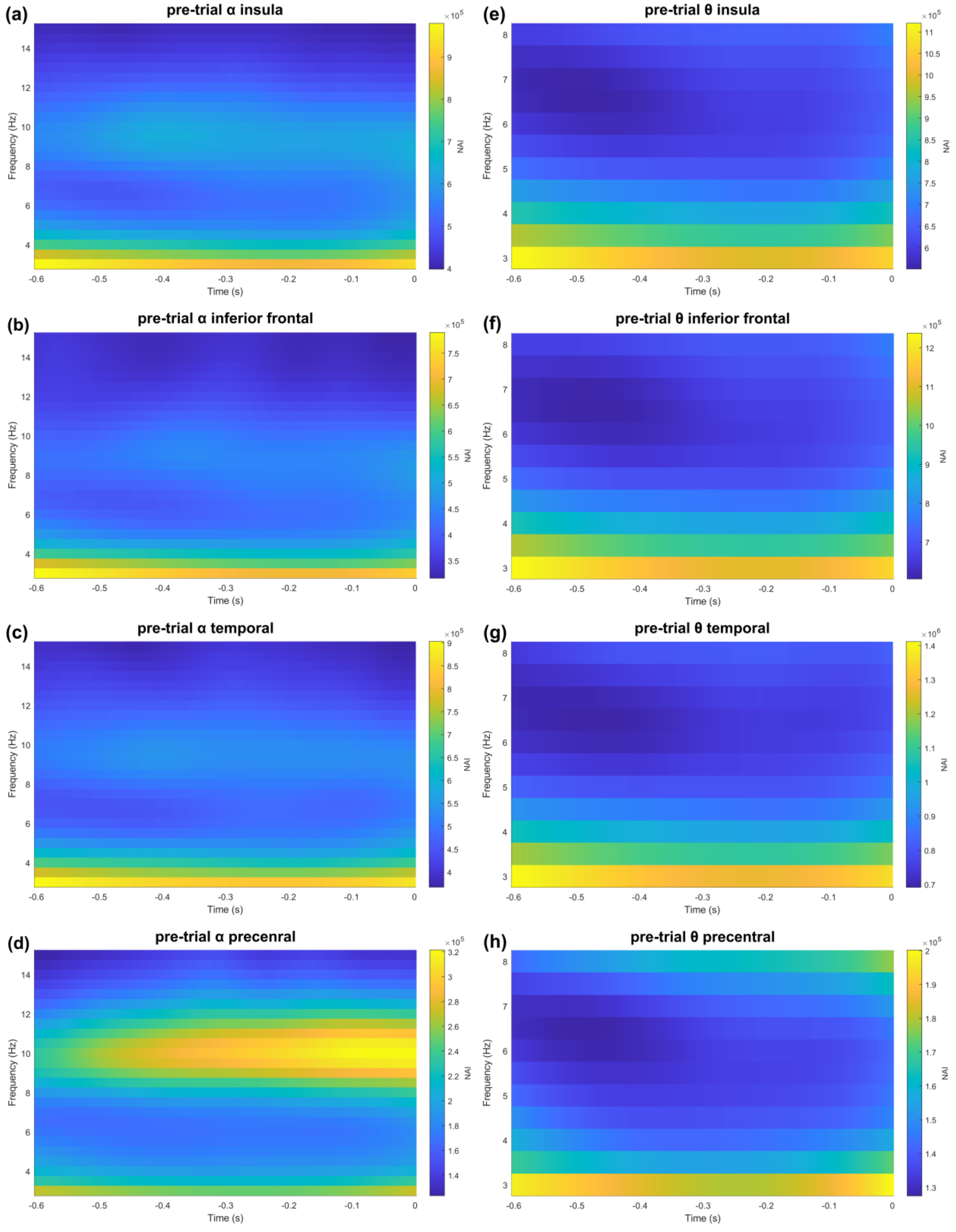

Supplemental figure 2.
Visualization of the time-frequency data after LCMV beamforming for each of the pre-trial clusters. Figure parts **(a-d)** show the time frequency representation for the four alpha-band clusters while **(e-h)** shows the time-frequency representation in the four theta-band pre-trial clusters. The x-axis represents the time before the onset of the stimulus (-0.6 to 0 s) while the y-axis represents the frequency. The color of the plot scales the magnitude of the neural activity index (NAI).

Supplemental table 1

Clusters as identified by the DBSCAN algorithm. Regions are labelled according to the AAL (automatic anatomical labelling) atlas (Tzourio-Mazoyer et al., 2002).

Frequency/time	Cluster	AAL regions	Voxels
Alpha pre-trial	1	Insula R	47
	2	Frontal Inf Orb R	54
	3	Temporal Pole Sup R	35
		Temporal Pole Mid R	08
		Temporal Sup R	06
	4	Precentral R	11
Alpha within-trial	1	Frontal Mid R	28
		Frontal Inf Tri R	16
Theta pre-trial	1	Insula R	23
	2	Frontal Inf Orb R	07
	3	Temporal Pole Sup R	06
	4	Precentral R	70
		Frontal Mid R	43
		Frontal Sup R	40
Theta within-trial	1	Paracentral Lobule L	44
	2	Cingulum Mid L	11
	3	Postcentral L	08
		Precentral L	05

Supplemental table 2

Mean R^2 for each pre-trial correlation matrix.

	α insula	α inferior frontal	α temporal	α precentral
θ insula	0.647	0.488	0.491	0.227
θ inferior frontal	0.522	0.688	0.611	0.398
θ temporal	0.338	0.451	0.584	0.235
θ precentral	0.387	0.284	0.275	0.287
